# Capsules Rheology in Carreau–Yasuda Fluids

**DOI:** 10.3390/nano10112190

**Published:** 2020-11-03

**Authors:** Alessandro Coclite, Giuseppe Maria Coclite, Domenico De Tommasi

**Affiliations:** 1 School of Engineering, Università della Basilicata, 85100 Potenza, Italy; 2 Dipartimento di Meccanica, Matematica e Management, 70126 Politecnico di Bari, Italy; giuseppemaria.coclite@poliba.it; 3 Dipartimento di Scienze dell’Ingegneria e dell’Architettura, 70126 Politecnico di Bari, Italy; domenico.detommasi@poliba.it

**Keywords:** immersed boundary method (IBM), dynamic forcing IBM, multi relaxation time (MRT), moving least squares, non-Newtonian rheology, particle margination

## Abstract

In this paper, a Multi Relaxation Time Lattice Boltzmann scheme is used to describe the evolution of a non-Newtonian fluid. Such method is coupled with an Immersed-Boundary technique for the transport of arbitrarily shaped objects navigating the flow. The no-slip boundary conditions on immersed bodies are imposed through a convenient forcing term accounting for the hydrodynamic force generated by the presence of immersed geometries added to momentum equation. Moreover, such forcing term accounts also for the force induced by the shear-dependent viscosity model characterizing the non-Newtonian behavior of the considered fluid. Firstly, the present model is validated against well-known benchmarks, namely the parabolic velocity profile obtained for the flow within two infinite laminae for five values of the viscosity model exponent, *n* = 0.25, 0.50, 0.75, 1.0, and 1.5. Then, the flow within a squared lid-driven cavity for *Re* = 1000 and 5000 (being *Re* the Reynolds number) is computed as a function of *n* for a shear-thinning (*n* < 1) fluid. Indeed, the local decrements in the viscosity field achieved in high-shear zones implies the increment in the local Reynolds number, thus moving the position of near-walls minima towards lateral walls. Moreover, the revolution under shear of neutrally buoyant plain elliptical capsules with different Aspect Ratio (*AR* = 2 and 3) is analyzed for shear-thinning (*n* < 1), Newtonian (*n* = 1), and shear-thickening (*n* > 1) surrounding fluids. Interestingly, the power law by Huang et al. describing the revolution period of such capsules as a function of the Reynolds number and the existence of a critical value, *Re*${}_{c}$, after which the tumbling is inhibited in confirmed also for non-Newtonian fluids. Analogously, the equilibrium lateral position $y_{eq}$ of such neutrally buoyant capsules when transported in a plane-Couette flow is studied detailing the variation of $y_{eq}$ as a function of the Reynolds number as well as of the exponent *n*.

## 1. Introduction

Nowadays, biological systems are triggering the interest of more and more scientists, highlighting the need of affordable analytical and numerical tools for describing processes in a wide range of spatial and temporal scales, within a number of competing biophysical effects. Without the conceit of being exhaustive, one can think at: the modeling of protein folding/unfolding when interacting with biological substrates [1,2,3,4]; the analysis of patterns induced by tissue growth [5] and detection of disease by tomography images [6,7]; nano-particles as promising mean in early-detection, treatment, and follow-up of cancer [8,9].

Specifically, nanomedicines have been widely accepted in the last few years as efficient carriers of therapeutics for patient- and disease-specific treatments [8,9]. For the specific release of drugs, two steps are required: nano-constructs accumulation into capillary peripheries of diseased tissue (*margination*) and firm adhesion to tissutal walls [10,11,12]. Indeed, particles’ non-Newtonian rheology plays a fundamental role in understanding specific mechanisms for predicting particle margination and thus for the rational design of nanopharmaceuticals [13,14,15,16]. Efficient computational techniques for biologically inspired problems are quickly gaining interest in the scientific community. Reliable numerical simulations of biological systems give fundamental insights for unraveling the physics behind such complex scenarios and, in turn, with dramatic reduction of the economic burden needed for experiments. In this context, due to the large number of parameters involved in the transport of platelets-like objects, computational methods are becoming of ever increasing interest [17,18].

In this work, a dynamic-Immersed–Boundary (IB) method is combined with a Multi-Relaxation–time Lattice Boltzmann (MRT-LB) scheme for describing the evolution of capsules transported in incompressible flows [19]. This method was extensively studied and validated by Coclite and collaborators [20,21,22,23,24,25]. Here, the authors propose an extension of such scheme to non-Newtonian fluids. Specifically, the forced Navier–Stokes equation is modeled through a two-dimensional MRT scheme with nine reticular velocities (D2Q9) in which the Guo forcing procedure is adopted [26]. Such forcing term accounts for spreading onto the lattice the total force exerted by the fluid: the body force due to the presence of an immersed body and the force induced by the adopted shear-dependent viscosity model. On one hand, no-slip boundary conditions on an immersed structures are imposed by a body-force accounting for the hydrodynamics force generated by the presence of the immersed body [19]. Immersed structures are transported as a collection of massive Lagrangian points connected by linear elements and their motion is obtained by solving the Euler–Newton equations for the center of mass and the revolution angle [27]. On the other hand, non-Newtonian effects on the fluid evolution are appointed through the shear-stress dependent force resulting from the local variation in the apparent viscosity regulated by the Carreau–Yasuda model for non-Newtonian fluids [28]. Commonly, non-Newtonian effects are accounted in such frameworks by considering the correlation between the relaxation time and the kinematic viscosity; thus, for each time step, the apparent viscosity field is computed and locally the relaxation time is obtained [29,30,31,32,33]. However, it is easy to recognize that such procedure may fall out of the stability range of relaxation times for the specific Lattice Boltzmann technique implemented during computations [34,35,36,37,38]. On the contrary, by using a forcing term to incorporate all of the non-Newtonian effects without any local variation of the relaxation time, the stability of the numerical scheme is granted [28]. Specifically, the proposed MRT-IB scheme presents two key features: on one side, as is widely known, MRT frameworks allow for accurately reconstructing incompressible flows characterized by a larger interval of Reynolds numbers with respect to Single Relaxation Time (SRT) formulation by overcoming SRT stability constraints without losing the ability of computing complex flows in the limit of small-but-non-null Reynolds numbers; on the other side, by accounting for the non-Newtonian effects through the shear-stress dependent force term without considering any variation of the relaxation time, the present formulation overcomes the second source of possible stability issues of the more classical SRT framework.

Firstly, the proposed model is validated by comparing the computed velocity profiles for a non-Newtonian fluid within two laminae driven by a constant pressure gradient with analytical predictions. Specifically, the plane-Pouseuille flow at Reynolds number (*Re*) 200 for a Carreau–Yasuda fluid with *n* = 0.25, 0.5, 0.75, 1.0, and 1.5 is computed. Moreover, this test is repeated on five different Eulerian meshes, and the second order accuracy of the present formulation is demonstrated. Then, the flow within a lid-driven cavity for *Re* = 1000 and 5000 when considering a shear-thinning fluid is analyzed. Interestingly, the minimum of the velocity profile taken at *x/L* = 0.5 in the near south-wall region displaces toward the wall for *n* < 1. The adopted model for the shear-dependent viscosity predicts the local decreasing of the kinematic viscosity as increases the local shear-rate (*vice versa* for *n* > 1). For lower values of *n* in the near-wall region, strong shear-strain is registered and thus the reduction of the local viscosity with, indeed, a local gain in the Reynolds number. This mechanism enhances the recirculation in the near-wall region and consequently the displacement of the velocity profile minimum. The dynamic behavior of elliptical particles immersed in a linear laminar flow is also studied for a shear-thinning (*n* < 1), Newtonian (*n* = 1), and shear-thickening (*n* > 1) fluids. Specifically, on one side, the tumbling motion of a neutrally buoyant capsule under shear is computed by considering two laminae countermoving with opposite velocity and the resulting revolution of a particle placed in the center of the domain is observed. This motion is analyzed by measuring the revolution period T as a function of the Reynolds number and in term of particle shape (elliptical particles with aspect ratio equal to 2 and 3) for *n* = 0.5, 1.0, and 1.5. The existence of a critical value for the Reynolds number *Re*${}_{c}$ is demonstrated for all of the investigated values of *n*. As per the lid-driven cavity test case, smaller (larger) values of the exponent *n* prescribe smaller (larger) Re${}_{c}$ for both particles, *AR* = 2 and 3. Analogously, this effect is due to the local variations in the apparent viscosity field induced by the local shear. On the other side, the scenario is slightly complicated by considering one of the two countermoving laminae (the bottom wall) with null velocity, in order to observe particles transport along with their rotation. As is well known, particles freely moving in a Couette flow would achieve an equilibrium lateral position, $y_{eq}$, depending on their shape and on the Reynolds number of the flow. In this work, we focus on the effect of the exponent *n* when considering an elliptical particle with *AR* = 2 in a Couette flow at *Re* ∈[50–200]. It is found that *n* dramatically influences the margination abilities of such particle for *Re* > 100 and that this effect is stronger for higher *Re* as well as for lower *n*.

This systematic analysis of the rheology of inertial particle immersed in non-Newtonian linear laminar flows provide valuable insights for the rational design on micro- and nano-particles for the specific delivery of pharmaceuticals. Specifically, the exponent *n* of the shear-dependent viscosity model plays a major role in particles’ margination abilities by moving upward or downward the critical Reynolds number inhibiting their tumbling motion as a function of *n*. The existence of such critical Reynolds number inhibiting the tumbling as well as the measure of the equilibrium lateral positions as a function of the Reynolds number were already pointed out for Newtonian fluids numerically and experimentally into several papers [39,40,41]. However, a thorough analysis of such phenomenon including non-Newtonian effects was missing. In a future paper, we plan to study in detail the system dynamics, when the elastic deformation of such capsules is allowed. To this scope, the capsules will be modeled as an elastic ring with extensional and bending stiffness that surrounds an incompressible fluid (see [42]).

## 2. Computational Method

### 2.1. Lattice Boltzmann Method

The forced Boltzmann equation is solved adopting the Multiple Relaxation Time (MRT) framework on a two-dimensional manifold [43]. Specifically, the evolution of the fluid is defined in terms of the set of nine discrete distribution functions obeying the Boltzmann equation and defined onto the velocity space, $\left[f_{i}\right],\left(i=0,\cdots ,8\right)$:

(1) $$f_{i}\left(\vec{x}+{\vec{e}}_{i}\Delta t,t+\Delta t\right)-f_{i}\left(\vec{x},t\right)\;=\;-M_{i,j}^{-1}\left(S_{ij}\left[m_{i}\left(\vec{x},t\right)-m_{i}^{eq}\left(\vec{x},t\right)\right]\right)+\left(1-S_{ij}\frac{\Delta t}{2}\right)m_{j}^{F}\left(\vec{x},t\right)\;,$$

in which $\vec{x}$ and *t* are the spatial and time coordinates, respectively; Δt is the time step; and $\left[{\vec{e}}_{i}\right],\left(i=0,...,8\right)$ is the set of *N* discrete velocities depicting the local discretization of the velocity space. On the two-dimensional square lattice with nine reticular velocities (D2Q9) [44], the set of discrete velocities is given by

(2) $${\vec{e}}_{i}=\left\{\begin{array}{cc} (0,0)\; & \;\mathrm{if}\;i\;=\;0\;,\\ \left(\cos\left(\frac{(i-1)\pi }{2}\right),\sin\left(\frac{(i-1)\pi }{2}\right)\right)\; & \;\mathrm{if}\;i\;=\;1-4\;,\\ \sqrt{2}\left(\cos\left(\frac{(2i-9)\pi }{4}\right),\sin\left(\frac{(2i-9)\pi }{4}\right)\right)\; & \;\mathrm{if}\;i\;=\;5-8\;. \end{array}\right.$$

$M_{i,j}$ ($M_{i,j}^{-1}$) is a matrix transforming the distribution functions $f_{i}$ into the moment vector $m_{i}\;=\;M_{ji}f_{j}$ ($f_{i}\;=\;M_{i,j}^{-1}m_{j}$):

(3) $$\bar{M}\;=\;\left|\begin{array}{ccccccccc} 1 & 1 & 1 & 1 & 1 & 1 & 1 & 1 & 1\\ -4 & -1 & -1 & -1 & -1 & 2 & 2 & 2 & 2\\ 4 & -2 & -2 & -2 & -2 & 1 & 1 & 1 & 1\\ 0 & 1 & 0 & -1 & 0 & 1 & -1 & -1 & 1\\ 0 & -2 & 0 & 2 & 0 & 1 & -1 & -1 & 1\\ 0 & 0 & 1 & 0 & -1 & 1 & 1 & -1 & -1\\ 0 & 0 & -2 & 0 & 2 & 1 & 1 & -1 & -1\\ 0 & 1 & -1 & 1 & -1 & 0 & 0 & 0 & 0\\ 0 & 0 & 0 & 0 & 0 & 1 & -1 & 1 & -1 \end{array}\right|\;.$$

$S_{i,i}$ is the relaxation times matrix. It is diagonal into the moment space, $S_{i,i}\;=\;diag\left(s_{0},s_{1},s_{2},s_{3},s_{4},s_{5},s_{6},s_{7},s_{8}\right)$, with $s_{i}$ the i-th relaxation time. $s_{0}$, $s_{3}$, and $s_{5}$ are set equal to 1 in order to ensure the perfect conservation of the corresponding macroscopical quantities, namely ρ, $\rho u_{x}$, and $\rho u_{y}$. Then, $s_{1}\;=\;s_{2}\;=\;1.4$ and $s_{6}\;=\;1.2$ while $s_{7}\;=\;s_{8}\;=\;s_{\nu }$ [43]. The kinematic viscosity of the flow is strictly related to $s_{\nu }$ as $\nu =c_{s}^{2}\left(\frac{1}{s_{\nu }}-\frac{\Delta t}{2}\right)$, being $c_{s}=\frac{1}{\sqrt{3}}$ the reticular speed of sound. The first two statistical moments of the distribution functions define the fluid density $\rho =\sum_{i}f_{i}$, and the momentum $\rho u=\sum_{i}f_{i}e_{i}+\frac{\Delta t}{2}f^{tot}$ while the pressure is obtained with the ideal equation of state $p=c_{s}^{2}\sum_{i}f_{i}$. The local equilibrium moments of the distribution functions $\left[m_{i}^{eq}\right]\left(i=0,...,8\right)$ read:

(4) $$m_{i}^{eq}\;=\;\left|\begin{array}{c} \rho \\ \rho (-2+3\left(u_{x}^{2}+u_{y}^{2}\right))\\ \rho (1-3\left(u_{x}^{2}+u_{y}^{2}\right))\\ \rho u_{x}\\ -\rho u_{x}\\ \rho u_{y}\\ -\rho u_{y}\\ \rho (u_{x}^{2}-u_{y}^{2})\\ \rho u_{x}u_{y} \end{array}\right|\;.$$

On the other hand, being $f^{tot}$, the total force acting on the fluid, the Guo forcing term [26,45] $\left[m_{i}^{F}\right]$(i=0,...,8) is given by:

(5) $$m_{i}^{F}\;=\;\left|\begin{array}{c} 0\\ \frac{1}{c_{s}^{4}}\left(f^{tot}\cdot u\right)\\ -\frac{1}{c_{s}^{4}}\left(f^{tot}\cdot u\right)\\ f_{x}^{tot}\\ -f_{x}^{tot}\\ f_{y}^{tot}\\ -f_{y}^{tot}\\ 2(f_{x}^{tot}u_{x}-f_{y}^{tot}u_{y})\\ f^{tot}\cdot u \end{array}\right|\;.$$

In the proposed scheme, the total force acting on the fluid is composed of a body force, $f^{ib}$ [19,20] accounting for the presence of immersed bodies and a shear-dependent force, $f^{nw}$, accounting for the non-Newtonian effects on the apparent viscosity of the fluid [28]. Finally, Dirichlet boundary conditions are imposed on external boundaries and treated with the known–velocity bounce back procedure by Zou and He [46].

### 2.2. Shear Induced Apparent Viscosity Treatment

***Carreau–Yasuda Model.*** In non-Newtonian fluids, the viscosity nonlinearly varies with the shear-rate. Specifically, this nonlinear relationship correlates the local kinematic viscosity with the shear strain tensor. In this paper, the Carreau–Yasuda model for the kinematic viscosity is adopted:

(6) $$\frac{\nu \left(\dot{\gamma }\right)-\nu_{\infty }}{\nu_{0}-\nu_{\infty }}\;=\;\left[1+{\left(\lambda \dot{\gamma }\right)}^{a}\right){]}^{\frac{n-1}{a}}\;.$$

**Figure 1:**
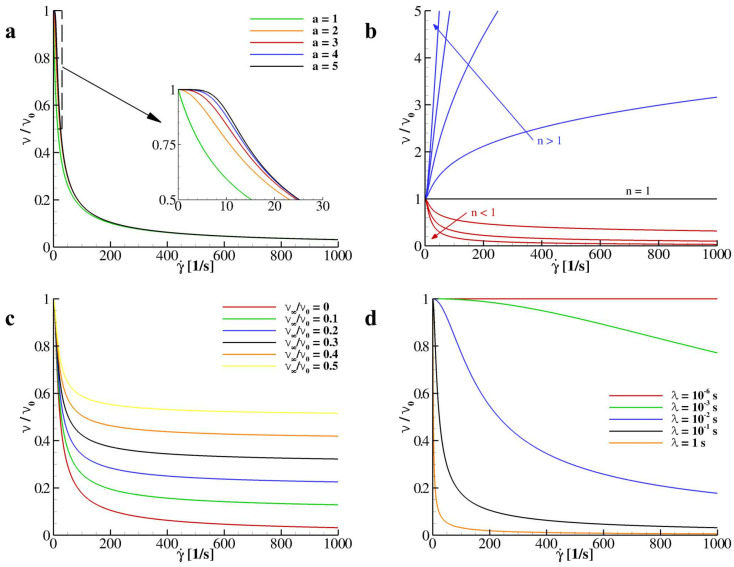
**Apparent viscosity as a function of the shear rate for different model parameters.** (**a**) Distribution of the viscosity for *a* = 1, 2, 3, 4, 5; *n* = 0.25; $\nu_{\infty }/\nu_{0}$ = 0; and λ=0.1. (**b**) Distribution of the viscosity for *a* = 2; *n* = 0.25, 0.50, 0.75, 1.0, 1.25, 1.5, 1.75, and 2.0; $\nu_{\infty }/\nu_{0}$ = 0; and λ=0.1. (**c**) Distribution of the viscosity for *a* = 2; *n* = 0.25; $\nu_{\infty }/\nu_{0}$ = 0, 0.1, 0.2, 0.3, 0.4, and 0.5; and λ=0.1. (**d**) Distribution of the viscosity for *a* = 2; *n* = 0.25; $\nu_{\infty }/\nu_{0}$ = 0; and $\lambda \;=\;10^{-6},\;10^{-3},\;10^{-2},\;10^{-1}$, and 1.0.

Specifically, this model correlates the local kinematic viscosity ν (measured in $m^{2}/s$) with the local shear-rate $\dot{\gamma }$ (measured in 1/s) through five constitutive parameters, four of which are material specific; $\nu_{0}$ and $\nu_{\infty }$ corresponding to the null- and infinite-shear values of the kinematic viscosity; *a* and *n* being two positive constants shaping the functional behavior of $\nu (\dot{\gamma })$; and the characteristic time scale λ that depends on the flow. *a* determines the position of the inflection point of the curve $\nu (\dot{\gamma })$ and how fast ν decreases (if *n* < 1) or increases (if *n* > 1) with $\dot{\gamma }$. However, by varying *a*, only in the low rates region $\dot{\gamma }\in \left[0,30\right]$ is registered variation in the distribution of $\nu /\nu_{0}$ as documented in Figure 1a. For this reason, in this paper, a is chosen to be equal to 2 as per the original Carreau model being $\nu /\nu_{0}$ almost unaffected by this parameter for higher values of $\dot{\gamma }$. On the other side, *n* returns whether the fluid exhibits a shear-thinning or shear-thickening behavior, while for n=1 the perfectly Newtonian behavior is recovered (see Figure 1b). Moreover, $\nu_{\infty }$ plays the role of limiter constant corresponding to the asymptotic value reached for $\dot{\gamma }\to \infty$ (see Figure 1c). As mentioned, λ is the characteristic time scale of the considered flow and is computed here through the Reynolds number, *Re* ($=\frac{u_{ref}l_{ref}}{\nu_{ref}}$). The reference viscosity $\nu_{ref}$ is chosen to be equal to $\nu_{0}$, while, depending on the flow analyzed, the reference velocity $u_{ref}$ and length $l_{ref}$ are set. Note that $\lambda^{-1}$ corresponds to the critical shear-rate at which shear-thinning or shear-thickening effects reveal. Specifically, low values of λ return a perfectly Newtonian behavior for the apparent viscosity while higher values give sudden variations of $\nu /\nu_{0}$ as depicted in Figure 1d.

***Lattice–Boltzmann method for shear-rate-dependent viscosity.*** To avoid any stability issues when locally varying the relaxation times as a consequence of the variation of the local viscosity as a function of $\dot{\gamma }$, the approach by Wang et al. [28] is adopted here. Instead of computing the local shear-dependent viscosity and transfer the relative variation to the corresponding relaxation times ($s_{7}\;=\;s_{8}\;=\;s_{\nu }$), in this approach, an equivalent forcing term is considered into the momentum equation by computing a shear-stress dependent force transferred to the distribution functions by the forcing term defined in Equation (5). The component α of the shear-dependent force for the Carreau–Yasuda model reads:

(7) $$f_{\alpha }^{nw}\;=\;2\left(\nu_{0}-\nu_{\infty }\right)\left\{{\left[1+{\left(\lambda \sqrt{2D_{\left|\right|}}\right)}^{2}\right]}^{\frac{n-1}{2}}-1\right\}\partial_{\alpha }S_{\alpha \beta },$$

where $S_{\alpha \beta }$ is the rate-of-strain tensor:

(8) $$S_{\alpha \beta }\;=\;\frac{1}{2}\left(\partial_{\alpha }u_{\beta }+\partial_{\beta }u_{\alpha }\right)\;,$$

through which $D_{\left|\right|}$ is computed being $D_{\left|\right|}=\sum_{\alpha \beta }S_{\alpha \beta }S_{\alpha \beta }$ and $\dot{\gamma }\;=\;\sqrt{2D_{\left|\right|}}$. The derivative of the rate-to-strain tensor is computed as follows [28]:

(9) $$\partial_{\alpha }S_{\alpha \beta }\;=\;\frac{c_{s}^{4}}{\Delta x}\sum_{i}e_{i,\alpha }S_{\alpha \beta }\left(x+e_{i}\Delta x\right)\;.$$

### 2.3. Immersed Boundary Treatment and Fluid–Structure Interaction

In this paper, a particle-based model is employed by coupling the Immersed-Boundary (IB) technique with an MRT-Lattice Boltzmann (MRT-LB) solver. The immersed body is a worm-like chain of nv vertices linked with nl linear elements, whose centroids are usually called *Lagrangian markers*. The IB procedure, extensively proposed and validated by Coclite and colleagues [19,20], is adopted here and a moving-least squares reconstruction is employed to exchange all MRT-LB distribution functions between the Eulerian lattice and the Lagrangian chain. Finally, the body force term in Equation (5), $f^{ib}$, is evaluated through the formulation by Favier et al. [47]. Moreover, particles dynamics are determined by the *dynamics IB* technique described in [19], using the solution of the Newton equation accounting for external stresses, with such particles being rigid structures. Then, no-slip boundary conditions are imposed using a weak coupling approach [20]. The external stresses exerted by the *l*-th linear element, namely pressure and viscous stresses, read:

(10) $$F_{l}^{p}\left(t\right)=\left(-p_{l}n_{l}\right)l_{l}\;,$$

(11) $$F_{l}^{\tau }\left(t\right)=\left({\bar{\tau }}_{l}\cdot n_{l}\right)l_{l}\;,$$

where ${\bar{\tau }}_{l}$ and $p_{l}$ are the viscous stress tensor and the pressure evaluated in the centroid of the element, respectively; $n_{l}$ is the outward normal unit vector while $l_{l}$ is its length. Pressure and velocity derivatives in Equations (10) and (11) are computed using a probe in the normal positive direction of each element, with the probe length being 1.2Δx [27]. Then, rigid motion is readily obtained by integrating all hydrodynamics stress contributions over the particles boundary and updating both linear and angular velocity in time as: $\dot{u}\left(t\right)\;=\;\frac{F^{tot}\left(t\right)}{m}$ and $\dot{\omega }\left(t\right)\;=\;\frac{M^{tot}\left(t\right)}{I}$, respectively, with *m* the immersed body mass, $F^{tot}$ and $M^{tot}$ the total force and moment acting on the particle, and *I* the body moment of inertia. Then, u(t) and ω(t) are derived through a second-order accurate finite difference scheme:

(12) $$u\left(t\right)\;=\;\frac{2}{3}\left(2u\left(t-\Delta t\right)-\frac{1}{2}u\left(t-2\Delta t\right)+\dot{u}\left(t\right)\Delta t\right)+O\left(\Delta t^{2}\right)\;,$$

(13) $$\omega \left(t\right)\;=\;\frac{2}{3}\left(2\omega \left(t-\Delta t\right)-\frac{1}{2}\omega \left(t-2\Delta t\right)+\dot{\omega }\left(t\right)\Delta t\right)+O\left(\Delta t^{2}\right)\;.$$

It should be noted that the present formulation is unconditionally stable for a small deformation of the capsule membrane and for small velocity variations applied, as previously demonstrated by Coclite et al. [19,20].

## 3. Results and Discussion

### 3.1. Flow within Two Laminae

**Figure 2:**
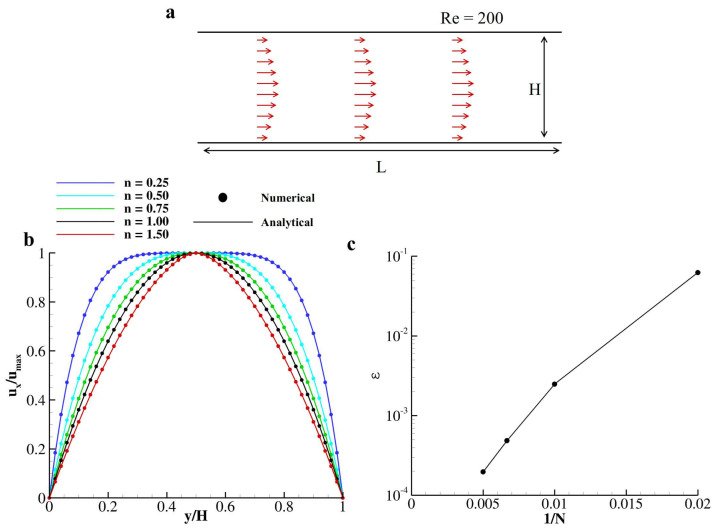
**Carreau–Yasuda flow within two parallel laminae at *Re* = 200.** (**a**) Schematic of the physical problem. (**b**) distribution of the normal velocity component for different values of *n* taken at x=0.5*L*; dots represent numerical predictions while lines are for the analytical solutions; (**c**) mesh refinement study on $u_{x}\left(y\right)$ profiles obtained with *n* = 0.75.

To validate the numerical scheme proposed in Section 2, the flow within two laminae is considered. Specifically, a rectangular domain by the height *H* = 200 Δx and length *L* = 6 *H* is adopted with periodic boundary conditions along the *x*-axis (Figure 2a). A plane Hagen–Poiseuille non-Newtonian flow is established by imposing a linear pressure drop Δp ($=\frac{8u_{max}^{2}}{H}\frac{\rho_{ref}x}{Re}$) along the channel as a function of the Reynolds number $Re\;=\;\frac{u_{max}H}{\nu_{0}}\;=\;200$, with *n* being the exponent of the viscosity model (see Equation (6)); $\nu_{0}$, $u_{max}$ and $\rho_{ref}$ the null shear kinematic viscosity, the velocity peak value, and the reference density, respectively. As previously discussed in Section 2.2, in the present paper, we adopt *a* = 2 and $\nu_{\infty }\;=\;0$. The proposed numerical scheme perfectly recovers the analytical predictions for the parabolic velocity profile for a non-Newtonian two-dimensional Poiseuille flow driven by a constant pressure gradient Δp,

(14) $$u\left(y\right)\;=\;\left(\frac{n}{n+1}\right)\left(\frac{\Delta p}{\rho_{ref}\nu_{0}}\right)\left[H/2^{\frac{n+1}{n}}-{\left(H/2-y\right)}^{\frac{n+1}{n}}\right]\;.$$

The obtained parabolic profile for *n* = 0.25, 0.5, 0.75, 1, and 1.5 is compared with analytical predictions, as reported in Figure 2b, returning a relative error of $1.968\times 10^{-4}$ with respect to the analytical solution. This test was repeated for *n* = 0.75 within five different Eulerian grid sizes (*H* = 50, 100, 150, 200, and 250 Δx) to prove the second-order accuracy of the present formulation. The *L*${}^{2}$-norm of the relative error with respect to the reference solution (obtained with *H* = 250 Δx), $\epsilon \;=\;||\frac{u_{x}-u_{x,H=250\Delta x}}{u_{x,H=250\Delta x}}{\left|\right|}^{2}$ is documented in Figure 2c. As reported, ϵ is well confined within 1% for H>50Δx.

### 3.2. Non-Newtonian Fluid in a Lid-Driven Cavity

**Figure 3:**
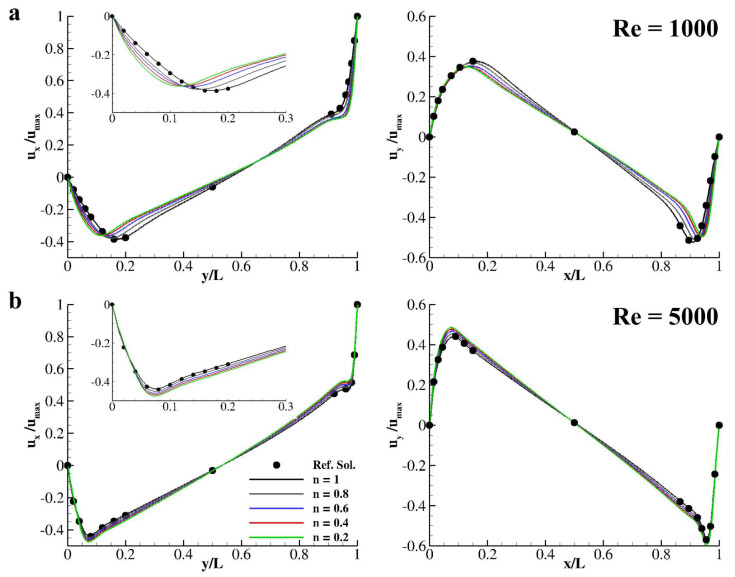
**Carreau–Yasuda fluid in a square lid-driven cavity.** Distribution of the *x* (left plot) and *y* (right plot) components of the velocity field taken at *x* = 0.5 *L* (left plot) and *y* = 0.5 *L* (right plot) for *n* = 0.2, 0.4, 0.6, 0.8, and 1.0 at *Re* = 1000 (**a**) and *Re* = 5000 (**b**). Close-ups emphasize the near-wall velocity profiles implying the central vortex displacements obtained lowering *n*. Dots represent numerical predictions obtained by Napolitano and Pascazio [48] while lines are for present model solutions.

To further validate the computational method for non-Newtonian fluids evolution, the flow in a lid-driven cavity is computed. Specifically, a box with side *L* = 500 Δx is considered. The wall at $y=y_{max}$ moves with velocity $u_{w}=\left(u_{max},0\right)$ with $u_{max}$ being the velocity peak value regulated by the Reynolds number *Re* = 1000 and 5000 (=$\frac{u_{max}L}{\nu_{0}}$); $\nu_{0}$ is the fluid null shear kinematic viscosity. The fluid inside the cavity is assumed and a shear-thinning fluid with *a* = 2, $nu_{\infty }\;=\;0$, and *n* ≤ 1. The resulting velocity distributions taken at *x* = 0.5 *L* and *y* = 0.5 *L* are reported in Figure 3a,b, for *Re* = 1000 and 5000, respectively. Indeed, *n* = 1 returns a perfectly Newtonian fluid and resembles the reference solution by Napolitano and Pascazio [48].

As demonstrated in Figure 1, by varying the exponent of the Carreau–Yasuda model for the apparent viscosity into the shear thinning region, namely n∈(0,1], an increase in the local Reynolds number is registered due to the local decrease of the kinematic viscosity. This local variation in the Reynolds number gives, in turn, a sensible variations in the velocity distributions taken at the two sections *x* = 0.5 *L* and *y* = 0.5 *L*. Specifically, for *Re* = 1000 and *n* = 1 in the proximity of the south-wall, a local minimum is found at *y/L* = 0.172 while, for *Re* = 5000, the minimum is at *y/L* = 0.075 (see Figure 3a,b left plots). Interestingly, for both Reynolds numbers, by varying *n*, the position of such minima move closer to the south wall. The position of this minima is reported as a function of *n* for *Re* = 1000 and *Re* = 5000 in Table 1.

### 3.3. Capsules Rotating under Shear

**Figure 4:**
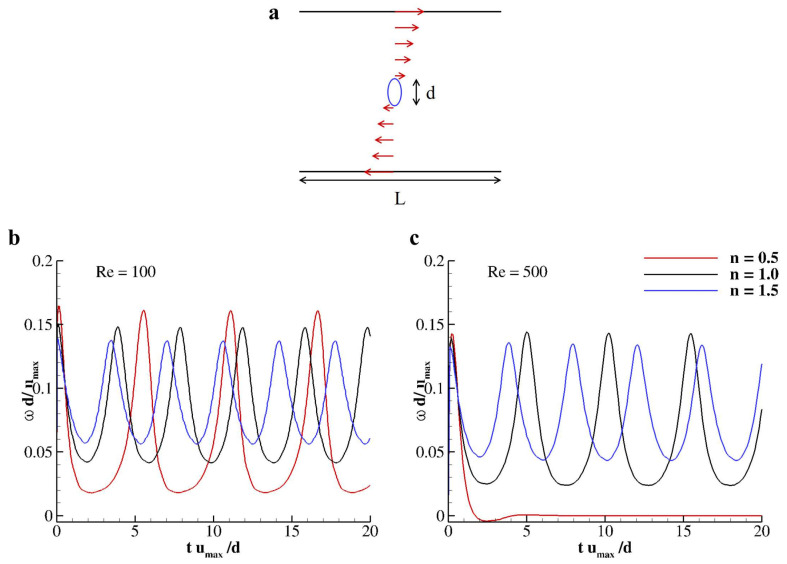
**Rigid elliptical particles rotating under shear in a Carreau–Yasuda fluid.** (**a**) schematic of the physical problem with characteristics dimensions and length. (**b**,**c**) distribution of the angular velocity for an elliptical particle with aspect ratio 2 for *n* = 0.5, 1, and 1.5 obtained for *Re* = 100 (**b**) and 500 (**c**).

The tumbling motion of a neutrally buoyant capsule in a shear flow is considered now. An elliptical particle with major axis *d* = 50 Δx and aspect ratio equal to 2 is placed in the center of a square domain with the side *L* = 10 *d* (see Figure 4a). Such particle is composed of 300 linear elements to have the ratio between the Lagrangian mesh and the Eulerian grid discretization correspond to 0.3 [20]. Top and bottom walls countermove with velocity $u_{max}$ while periodic boundary conditions are imposed along *x*. The Reynolds number of the flow is defined as *Re* = $\frac{4\gamma d^{2}}{\nu_{0}}$, with $\gamma \;=\;\frac{u_{max}}{1/2L}$ being the shear rate and $\nu_{0}$ the null-shear kinematic viscosity [39]. The fluid into the square cavity is considered as shear-thinning (*n* = 0.5), Newtonian (*n* = 1) and as shear-thickening (*n* = 1.5) and the revolution period as a function of the Reynolds number is investigated. As already pointed out in several papers [40,49,50], a particle freely moving in a shear-flow would tumble with a period that depends on the Reynolds number of the established flow. Firstly, the revolution of the elliptical particle is studied for *Re* = 100 and 500 as a function of *n* (= 0.5, 1.0, and 1.5). As depicted in Figure 4b, the particle immersed in a shear flow at *Re* = 100 revolt with three different periods when varying *n*, specifically, *T* = 5.5753, 4.0046, and 3.5972 for *n* = 0.5, 1.0, and 1.5, respectively. The revolution period increases when decreasing *n* as well as the peak angular velocity being $\omega d/u_{max}$ = 0.1607, 0.1476, and 0.1370 for *n* = 0.5, 1.0, and 1.5, respectively. In the same fashion, for *Re* = 500, the particle is able to maintain its tumbling motion only for *n* = 1 and 1.5, while for *n* = 0.5 the motion is inhibited and the particle settle with an angle with the horizon $\theta_{c}\;=\;0.1898\;\pi$ (see Figure 4c).

**Figure 5:**
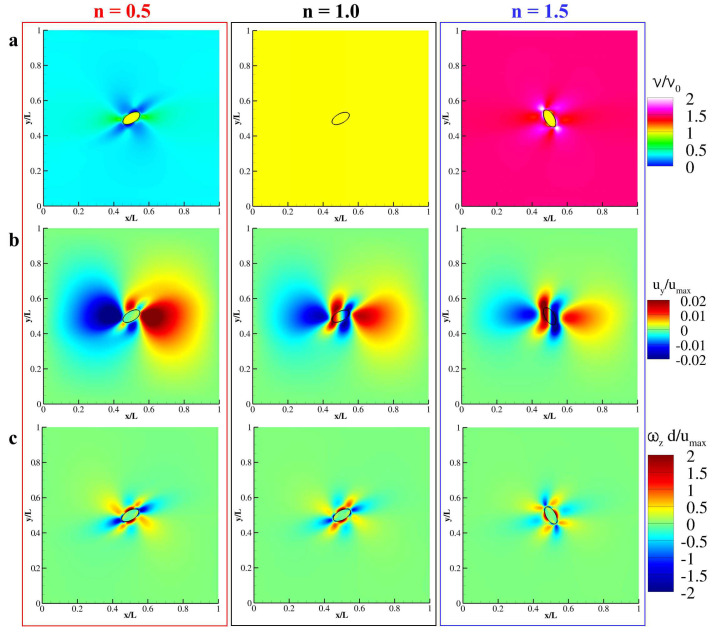
**Contour plot of conserved thermodynamical quantities at *Re* = 500.** Contour plot of the apparent viscosity (**a**), the y-component of the velocity (**b**) and out-of-plane vorticity (**c**) obtained for *n* = 0.5, 1, and 1.5 at *Re* = 500.

The contour plot of the computed apparent viscosity is detailed in Figure 5a (left) for *n* = 0.5 and clearly demonstrate that in the higher shear zones (i.e., the near-particle region) the apparent viscosity of the flow dramatically drops below 1 locally increasing the Reynolds number. On the other hand, for *n* = 1.5, the same region is interested in a dramatic increase of the apparent viscosity, thus reducing the local Reynolds number (see Figure 5a (right)). Indeed, for *n* = 1, no variation of $\nu /\nu_{0}$ is observed with the fluid being reduced to a Newtonian fluid (see Figure 5a (center)). Interestingly, the contour plots of $u_{y}/u_{max}$ present non-null velocity field of the fluid enclosed by the particle for *n* = 1 and 1.5, while, for *n* = 0.5, the internal fluid is the rest with the particle being blocked by the flow (see Figure 5b). Note that the outer velocity field for *n* = 0.5 is completely symmetrical with respect to the major axis of the capsule giving zero angular velocity on its boundary. Qualitatively, by seeing at the out-of-plane vorticity $\omega_{z}d/u_{max}$, documented in Figure 5c, the size and the intensity of the positive-vorticity zones are balanced by the size and the intensity of the negative-vorticity zones only for *n* = 0.5, while this equilibrium is unbalanced for *n* = 1 and 1.5. Thus, the particle is forced to tumble for *n* = 1 and 1.5 accordingly to $\omega_{z}d/u_{max}$ trying to nullify the angular slip velocity [20].

**Figure 6:**
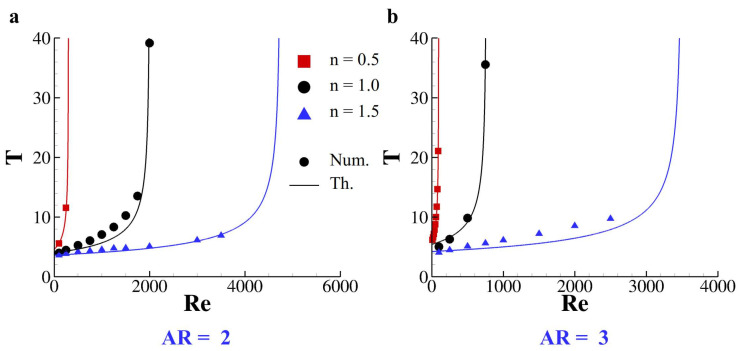
**Revolution period of elliptical particles in a Carreau–Yasuda fluid.** Revolution period as a function of the Reynolds number for elliptical particles with aspect ratio 2 (**a**) and 3 (**b**) rotating in a a Carreau–Yasuda fluid with *n* = 0.5, 1.0, and 1.5. Dots are for present model solutions while solid lines represent analytical predictions.

This tumbling motion presents a critical value for the Reynolds number (*Re*${}_{c}$) after which the particle stops in a fixed angular position. Specifically, the tumbling period *T* as a function of the Reynolds number follows a scaling law that reads: $T\;=\;c{\left(Re_{c}-Re\right)}^{-1/2}$ with *c* a positive constant [39,40]. Indeed, the values of $Re_{c}$ and *c* depend on the particle shape and density, on the surrounding fluid characteristics and on the blockage ratio. Here, we investigate the role of *n* for neutrally buoyant elliptical particles with aspect ratio (*AR*) 2 and 3. The revolution period of both particles (*AR* = 2 and 3) is measured as a function of the Reynolds number and the existence of *Re*${}_{c}$ is confirmed for *n* = 0.5, 1.0 and 1.5 (see Figure 6). The values of *c* and *Re*${}_{c}$ are tabulated for both particle in Table 2. Indeed, for lower values of *n*, lower critical Reynolds numbers are registered for both particles even if the more elongated particle (*AR* = 3) presents lower *Re*${}_{c}$ with respect to the ellipse with *AR* = 2 regardless from *n*.

### 3.4. Transport of Rigid Capsules in a Shear-Thinning Couette Flow

**Figure 7:**
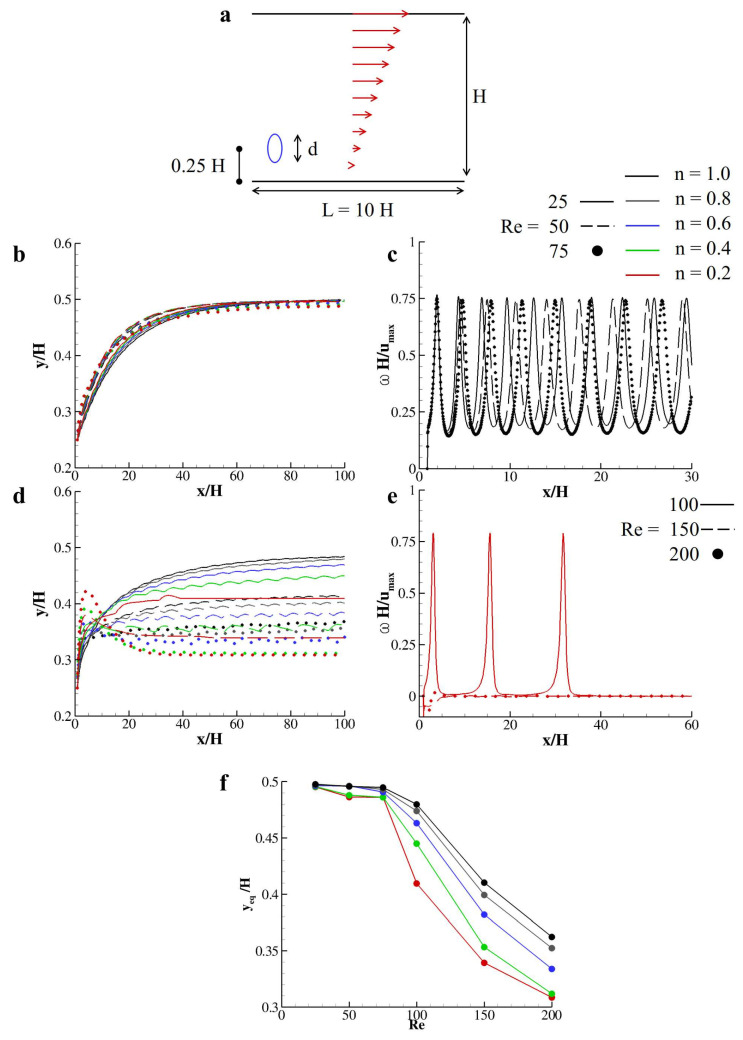
**Particles margination in a non-Newtonian Couette flow.** (**a**) schematic of the physical problem with reference lengths; (**b**) trajectory of a particle navigating a linear laminar flow for *Re* = 25, 50, and 75 as a function of *n*; (**c**) distribution of the angular velocity over the horizontal coordinate for *Re* = 25, 50, and 75 and *n* = 1; (**d**) trajectory of a particle navigating a linear laminar flow for *Re* = 100, 150, and 200 as a function of *n*; (**e**) distribution of the angular velocity over the horizontal coordinate for *Re* = 100, 150, and 200 and *n* = 0.2; (**f**) equilibrium lateral positions as a function of the Reynolds number for *n* = 0.2, 0.4, 0.6, 0.8, and 1.

The lateral displacement of a particle released in a non-Newtonian Couette flow established in a rectangular duct is considered now. The computational domain corresponds to a rectangular duct with the top wall moving with velocity $u_{max}$ regulated by the Reynolds number *Re* = $\frac{u_{max}d}{\nu_{0}}$, with *d* = 50 Δx being the particle characteristic length and $\nu_{0}$ the null-shear value of the kinematic viscosity. The particle is chosen as an elliptical capsule with aspect ratio 2 and is placed, initially at the rest, at *y* = 0.25 *H* with *H* = 4 *d* and *d* the major axis of the ellipse. Periodic boundary conditions are imposed at the sections *x* = 0 and *x* = *L* = 10 *H* (see Figure 7a). In this context, particles’ lateral drifting (*margination*) depends on the exerted lift composed by: (i) the inertial lift due to shear slip; (ii) the lift due to rotational slip; and (iii) lubrication effect due to the presence of the bottom wall [20]. The cooperation between these three components shows that particles would be dislodged by their releasing position and move laterally to find an equilibrium position as a function of the Reynolds number. The aim of the present study is to understand the influence of *n* in the equilibrium position of such particles when considering a shear-thinning fluid within the two laminae. First, the particle journey is detailed for moderate Reynolds numbers (Figure 7b). Specifically, for all values of *n*, the particle reaches exactly the channel midway for *Re* = 25, while, for *Re* = 50 and 75, the equilibrium positions slightly differ, although they are all around ≈0.48 *H*. During this journey, the particles rotate with an angular velocity that essentially depends on Re. The distributions of $\omega H/u_{max}$ for *Re* = 25, 50, and 75 are depicted in Figure 7c, only *n* = 1 having all of the distributions almost overlapped. For moderate-to-high Reynolds numbers, the overall picture changes and the particles settle to significantly different equilibrium lateral positions as a function of *Re* and *n*. The trajectory of such particles for *Re* = 100, 150, and 200 as a function of *n* is reported in Figure 7d. The tumbling motion of the particle may stop during their journey and the lift component due to the rotational slip becoming null. This implies that the particle would settle to a specific equilibrium position that is determined only by the lubrication and shear slip effects. In Figure 7e, this effect is demonstrated for *n* = 0.2. For *Re* = 100, the particle starts to tumble and moves from the releasing position 0.25 *H* to 0.37 *H* traveling from 0.9 *x/H* to ≈10 *x/H*. Then, it stays parallel to the south wall while navigating the duct from 10 *x/H* to 14.54 *x/H* and a second rotation happens pushing the particle to 0.390 *y/H*. As soon as the particle reaches the equilibrium position *y*${}_{eq}$ = 0.409 *y/H*, the tumbling motion is inhibited (see the red solid line in Figure 7d,e). The equilibrium position of such particle as a function of *Re* for all the investigated values of *n* is reported in Figure 7f, demonstrating how for a shear-thinning fluid the critical Reynolds number that inhibits the tumbling motion of immersed bodies is as small as the shear-thinning effect is strong (as *n* is smaller than 1). Note that, for the presented computations, the tumbling motion was inhibited only for *n* = 2 at *Re* = 100, 150, and 200 and for *n* = 0.4 for *Re* = 200. All of the other parameters’ combinations present both the lateral dislodging and the tumbling motions so that, for such particles, the journey continues with their center of mass at the equilibrium quote while revolting around their own axis.

## 4. Conclusions

The rheology of neutrally buoyant capsules immersed in Carreau–Yasuda fluids is considered and critically analyzed in this paper. The incompressible forced Navier–Stokes equation is modeled through a multi relaxation time lattice Boltzmann scheme in which a forcing term is demanded to spread to the distribution functions: (i) the body force due to the presence of an immersed body; and (ii) the force induced by the shear-dependent viscosity model characterizing the non-Newtonian behavior of the considered fluid. Such forcing term is included as an additional factor into the momentum equation and is appointed to spread the total force exerted by the fluid into the Eulerian grid nodes. In this work, such force is composed by two parts: on one side, the no-slip boundary conditions on an immersed structure are imposed by a body-force accounting for the hydrodynamics force generated by the presence of the immersed body; on the other side, the shear-stress dependent force due to the local variation in the apparent viscosity regulated by the Carreau–Yasuda model for non-Newtonian fluids.

The proposed model was validated against two well-known benchmark tests: firstly by measuring the velocity profile obtained when considering the flow within two laminae at Reynolds number equal to 200 as a function of the exponent of the Carreau–Yasuda model (*n* = 0.25, 0.50, 0.75, 1.0, and 1.5); then, the flow in a lid-driven cavity for two different Reynolds numbers (*Re* = 1000 and 5000) for a shear-thinning fluid characterized by five different values of *n*. In the latter case, the velocity profile taken at *x/L* = 0.5 presents a minimum located near the southern wall, such minimum moves approaching the wall as *n* decreases, thus mimicking the effect of an increase in the Reynolds number of the flow.

The rheology of elliptical particles with Aspect Ratio (*AR*) equal to 2 and 3 is analyzed by considering two diverse scenarios. On one hand, in order to isolate the tumbling motion and inhibit the translation over the computational domain, the revolution under shear of such capsules is considered by posing a single capsule in the middle of a square domain with countermoving top and bottom walls. The tumbling motion is observed and the revolution period T is measured as a function of *Re* for shear-thinning (*n* < 1), Newtonian (*n* = 1), and shear-thickening (*n* > 1) surrounding fluids. The critical Reynolds number after whom the rotational motion stops is determined for all cases. Interestingly, smaller (larger) values of the exponent *n* prescribe smaller (larger) *Re*${}_{c}$ for both particles, *AR* = 2 and 3. This effect is due to the local variations in the apparent viscosity field induced by the local shear. Analogously, the equilibrium lateral position $y_{eq}$ of such neutrally buoyant capsules when transported in a plane-Couette flow is studied detailing the variation of $y_{eq}$ as a function of the Reynolds number for shear-thinning fluids. Specifically, it is found that the exponent *n* dramatically increase the margination abilities of such particles. In fact, for moderate Reynolds numbers (*Re* ≈ 100–200), the equilibrium lateral position corresponds to about particle characteristic dimension $y_{eq}\approx 0.3$ H.

Collectively, these data represent specific insights for the scientific community investing efforts to the rational design of micro- and nano-particles as drug carriers. Specifically, the rheology of micro-capsules immersed in non-Newtonian fluids and the analysis of their marginating dynamics would help the community to precisely tailor particle shape in order to specifically determine their journey into human micro–circulation. 

## Figures and Tables

**Table 1:** Position of the minimum located into the near south-wall region for the velocity profiles taken the section *x* = 0.5 *L* as a function of *n*. *Re* = 1000 (**a**) and 5000 (**b**).

	(**a**) *Re* = 1000
***n***	**South-Wall Min. [y/L]**
1.0	0.172
0.8	0.150
0.6	0.138
0.4	0.122
0.2	0.114
	(**b**) *Re* = 5000
***n***	**South-Wall Min. [**y**/L]**
1.0	0.075
0.8	0.074
0.6	0.073
0.4	0.072
0.2	0.072

**Table 2:** Parameter of the fitting scaling law for the revolution period as a function of *n* for rigid elliptical particles with aspect ratio 2 (**a**) and 3 (**b**).

(**a**) *AR* = 2		
***n***	***c***	**Re** ${}_{c}$
0.5	80	300
1.0	180	2005
1.5	250	4750
(**b**) *AR* = 3		
***n***	***c***	**Re** ${}_{c}$
0.5	58	97
1.0	150	765
1.5	250	3500
